# Refinements for Bragg coherent X-ray diffraction imaging: electron backscatter diffraction alignment and strain field computation

**DOI:** 10.1107/S1600576722007646

**Published:** 2022-09-06

**Authors:** David Yang, Mark T. Lapington, Guanze He, Kay Song, Minyi Zhang, Clara Barker, Ross J. Harder, Wonsuk Cha, Wenjun Liu, Nicholas W. Phillips, Felix Hofmann

**Affiliations:** a Department of Engineering Science, University of Oxford, Parks Road, Oxford OX1 3PJ, UK; b Department of Materials, University of Oxford, Parks Road, Oxford OX1 3PH, UK; c Advanced Photon Source, Argonne National Laboratory, Argonne, IL 60439, USA; d Paul Scherrer Institut, 5232 Villigen PSI, Switzerland; SLAC National Accelerator Laboratory, Menlo Park, USA

**Keywords:** Bragg coherent X-ray diffraction imaging, electron backscatter diffraction, strain calculation, phase interpolation, crystal orientation

## Abstract

Bragg coherent X-ray diffraction imaging (BCDI) allows the 3D measurement of lattice strain along the scattering vector for specific microcrystals. If at least three linearly independent reflections are measured, the 3D variation of the full lattice strain tensor within the microcrystal can be recovered. However, this requires knowledge of the crystal orientation, which is typically attained via estimates based on crystal geometry or synchrotron microbeam Laue diffraction measurements. Presented here is an alternative method to determine the crystal orientation for BCDI measurements using electron backscatter diffraction (EBSD) to align Fe–Ni and Co–Fe alloy microcrystals on three different substrates. The orientation matrix is calculated from EBSD Euler angles and compared with the orientation determined using microbeam Laue diffraction. The average angular mismatch between the orientation matrices is less than ∼6°, which is reasonable for the search for Bragg reflections. The use of an orientation matrix derived from EBSD is demonstrated to align and measure five reflections for a single Fe–Ni microcrystal via multi-reflection BCDI. Using this data set, a refined strain field computation based on the gradient of the complex exponential of the phase is developed. This approach is shown to increase accuracy, especially in the presence of dislocations. The results demonstrate the feasibility of using EBSD to pre-align BCDI samples and the application of more efficient approaches to determine the full lattice strain tensor with greater accuracy.

## Abstract 2

A novel and accurate method to align crystalline samples for Bragg coherent X-ray diffraction imaging using electron backscatter diffraction is presented. An efficient approach using the complex exponential of the phase to calculate the strain is also implemented, for greater accuracy in resolving crystal defects.

## 1. Introduction

Bragg coherent X-ray diffraction imaging (BCDI) allows 3D nanoscale strain measurements, with a typical spatial resolution of a few tens of nanometres and a strain resolution of the order of ∼2 × 10^−4^ (Hofmann *et al.*, 2017*b* ▸). BCDI has been applied to study crystal defects and lattice strain in a variety of materials, including noble metals (Robinson *et al.*, 2001 ▸), alloys (Kawaguchi *et al.*, 2021 ▸), geological compounds (Yuan *et al.*, 2019 ▸), semiconductors (Lazarev *et al.*, 2018 ▸) and functional materials (Dzhigaev *et al.*, 2021 ▸). An advantage of using BCDI is the ability to study 3D volumes up to 1 µm in size under ambient conditions. This has enabled BCDI to become an essential tool for probing how lattice strains evolve in *in situ* and *operando* studies, for example in battery charging (Singer *et al.*, 2018 ▸), thermal diffusion (Estandarte *et al.*, 2018 ▸), dissolution (Clark *et al.*, 2015 ▸) and catalytic oxidation (Carnis *et al.*, 2021 ▸).

BCDI involves fully illuminating a crystalline sample with a coherent X-ray beam and positioning the diffractometer such that the Bragg condition is met for a specific *hkl* reflection. The outgoing wavevector produces a diffraction pattern that is collected on a pixellated area detector positioned in the far field (Fraunhofer regime). By rotating the sample through the Bragg condition, a 3D coherent X-ray diffraction pattern (CXDP) is recorded as different parts of the 3D Bragg peak sequentially intersect the Ewald sphere in reciprocal space, which is projected onto the detector. If the CXDP is oversampled by at least twice the Nyquist frequency (Sayre, 1952 ▸), iterative phase retrieval algorithms can be used to recover the phase (Fienup, 1982 ▸). The amplitude and phase in reciprocal space are related to the real-space object via an inverse Fourier transform (Miao & Sayre, 2000 ▸) followed by a space transformation from detector conjugated space to orthogonal laboratory or sample space (Yang *et al.*, 2019 ▸; Maddali *et al.*, 2020 ▸; Li *et al.*, 2020 ▸). The real-space amplitude ρ(**r**), where **r** is the position vector, is proportional to the effective electron density of the crystalline volume associated with the particular crystal reflection. The real-space phase ψ(**r**) corresponds to the projection of the lattice displacement field **u**(**r**) onto the Bragg vector **Q**_*hkl*_ of a specific *hkl* crystal reflection,

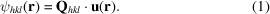




Since the development of BCDI in the early 2000s, most experiments have featured the measurement of a single reflection, providing only one component of the strain tensor. However, the analysis of a single strain component can be ambiguous as different information is obtained for different reflections (Yang *et al.*, 2021 ▸). If at least three linearly independent reflections are measured, the full 3D strain tensor can be calculated. Before 2017, only three experiments (Beitra *et al.*, 2010 ▸; Newton *et al.*, 2010 ▸; Ulvestad *et al.*, 2015 ▸) reported measuring more than one reflection on a single crystal. This is not surprising, as multi-reflection BCDI (MBCDI) experiments require prior knowledge of the crystal orientation (Newton *et al.*, 2010 ▸) or the scanning of extensive volumes of reciprocal space until two reflections are found, upon which further reflections can then be located.

The development of a microbeam Laue X-ray diffraction pre-alignment procedure in 2017 (Hofmann *et al.*, 2017*a* ▸) enabled the direct determination of the crystal orientation matrix, such that crystals could be reliably pre-aligned for MBCDI. Recently, a double-bounce Si(111) monochromator that allows Laue X-ray diffraction to be performed has been commissioned on the BCDI beamline 34-ID-C at the Advanced Photon Source (APS), Argonne National Laboratory, USA (Pateras *et al.*, 2020 ▸). Another method to determine the orientation of a sample is by indexing pole figures (Richard *et al.*, 2018 ▸), but this method requires a Bragg peak with known Miller indices to be found. The indexing is performed using texture analysis and relies on the samples being well faceted to produce truncation rods in reciprocal space that are perpendicular to the facet surfaces. These pre-alignment protocols have not only led to the increased popularity of MBCDI for determination of the full strain tensor with respect to an arbitrary reference (Yang *et al.*, 2022 ▸; Hofmann *et al.*, 2017*b* ▸, 2018 ▸, 2020 ▸; Phillips *et al.*, 2020 ▸) but also enabled simultaneous multi-Bragg-peak phase retrieval procedures to increase reconstruction quality (Newton, 2020 ▸; Gao *et al.*, 2021 ▸; Wilkin *et al.*, 2021 ▸).

Here we present an alternative method of pre-determining crystal orientation for MBCDI alignment without relying on synchrotron X-rays. We use electron backscatter diffraction (EBSD) to determine the orientation (Adams *et al.*, 1993 ▸) of randomly oriented Fe–Ni and Co–Fe microcrystals on three different sapphire substrates. EBSD instruments are much more widespread and accessible than synchrotron instruments and can be used as a valuable pre-screening tool for BCDI. EBSD measurements can produce 2D orientation maps with a high spatial resolution of ∼10 nm, thus enabling the selection of specific crystals with particular orientations or features such as twin domains. This allows the user to preserve synchrotron beamtime for BCDI measurements rather than performing pre-orientation measurements and analysis on the beamline. We compare orientation matrices found by EBSD with those measured by microbeam Laue diffraction and the ultimately measured reflection positions in MBCDI. Using the pre-determined EBSD orientation matrix, we measured five crystal reflections for an Fe–Ni microcrystal (Fig. 7, Section 3.2) and determined its full strain and rotation tensors with respect to the average structure of the crystal. We also implement an alternative approach using the complex component of the phase, rather than the phase alone, for the efficient calculation of the phase derivatives required for the strain tensor determination and the more accurate interpolatation of the recovered phase to sample coordinates.

## 2. Experimental methodology

### 2.1. Microcrystal fabrication

**Figure 1:**
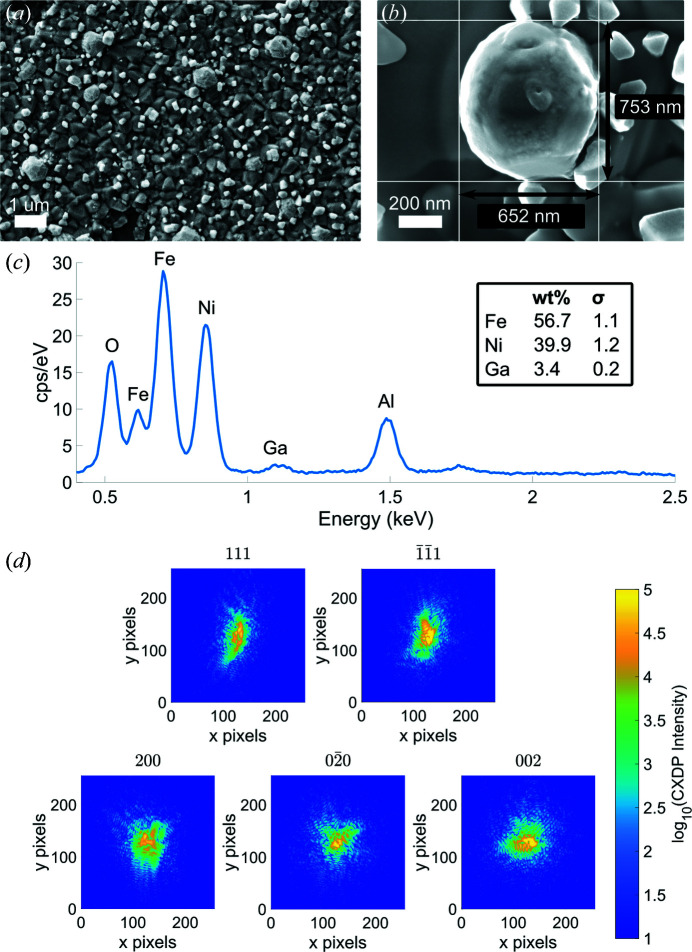
(*a*) Dewetted Fe–Ni alloy microcrystals on a sapphire substrate. (*b*) Fe–Ni microcrystal 1B is used for the computation of strain and rotation tensors. (*c*) The EDX spectrum for crystal 2B on the substrate, which is similar across all crystals on the substrate. The *L* lines for the most pronounced elements in the crystal are indicated. The composition excludes the Al and O substrate peaks. The Ga impurity is due to FIB milling around the crystal vicinity (Hofmann *et al.*, 2017*b* ▸). (*d*) Central slices of the CXDPs for each reflection, measured for crystal 1B.

Samples were produced by sputter deposition of a thin film onto a single-crystal sapphire wafer (C-plane orientation). One substrate with a film thickness of 375 nm was produced for the Fe–Ni microcrystals. It was dewetted in a vacuum furnace purged with a gas mixture of 5% hydrogen, balance argon, at 1523 K for 24 h. The resulting crystals exhibit a face-centred cubic (f.c.c.) structure, range from 0.5 to 1.5 µm in size [Fig. 1 ▸(*a*)] and adhere to the substrate surface. The substrate was cleaved to make substrates 1 and 2, both containing Fe–Ni microcrystals. Substrate 3 contained Co–Fe microcrystals that were produced in a similar way. The procedure and details for substrate 3 can be found elsewhere (Yang *et al.*, 2022 ▸).

**Figure 2:**
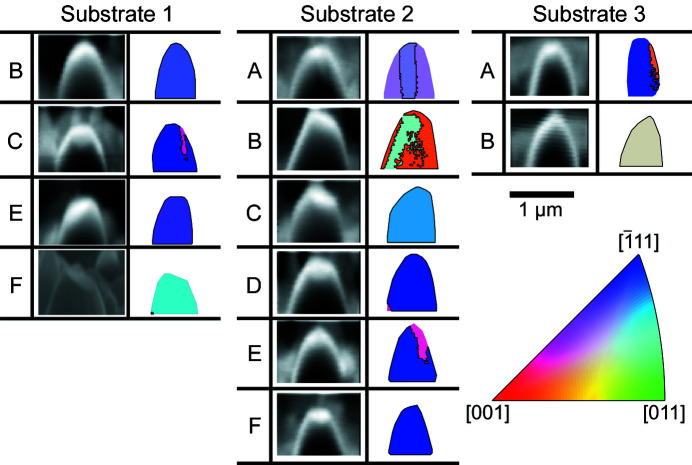
SEM images and inverse pole figure (IPF) maps for each crystal from EBSD. The colour coding shows the out-of-plane crystal orientation. The maps allow for different grains to be identified. For each crystal, the region surrounded by thick black lines corresponds to the grain orientation used for the computation of the orientation matrix. For some samples (*i.e.* 1F and 3A) only a small region on the IPF map corresponds to the grain measured at the synchrotron. EBSD samples the orientation of a cylindrical volume with a height up to 40 nm below the surface (Dingley, 2004 ▸).

Each substrate was coated with 10 nm of amorphous carbon via thermal evaporation using a Leica ACE600 coater to assist with scanning electron microscopy (SEM) imaging. To facilitate reliable measurement of multiple reflections from a specific microcrystal, a ZEISS NVision 40 Ga focused ion beam (FIB) instrument was used to remove the surrounding crystals within a 40 µm radius using currents from 6 nA to 150 pA and an acceleration voltage of 30 kV. Only SEM imaging was used to position the FIB milling scans to prevent large lattice strains caused by FIB imaging (Hofmann *et al.*, 2017*b* ▸). The isolated crystals on each substrate are shown in Fig. 2 ▸. Crystal 1B [Fig. 1 ▸(*b*)] was used for the computation of the strain and rotation tensors (Fig. 7).

**Figure 3:**
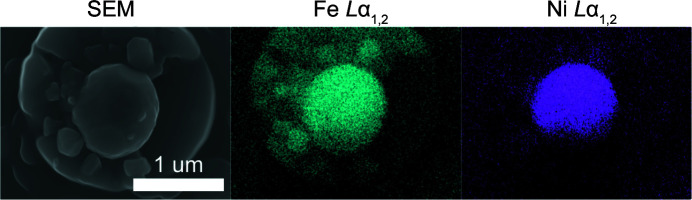
EDX elemental analysis maps of an Fe–Ni crystal, showing the homogeneous elemental distribution of crystal 2B corresponding to the SEM spectrum in Fig. 1 ▸(*c*). The images show the 2D signals for the primary Fe–Ni emission lines.

Energy-dispersive X-ray spectroscopy (EDX) was used to determine the elemental composition of each crystal [Fig. 1 ▸(*c*)]. EDX showed a homogeneous distribution of all elements throughout the dewetted crystals (Fig. 3 ▸). EDX was performed on a ZEISS Merlin instrument using an Xmax 150 detector (Oxford Instruments) with an elliptical region encapsulating crystal 2B on the substrate for 16 s with an accelerating voltage of 10 kV.

### 2.2. Electron backscatter diffraction

**Figure 4:**
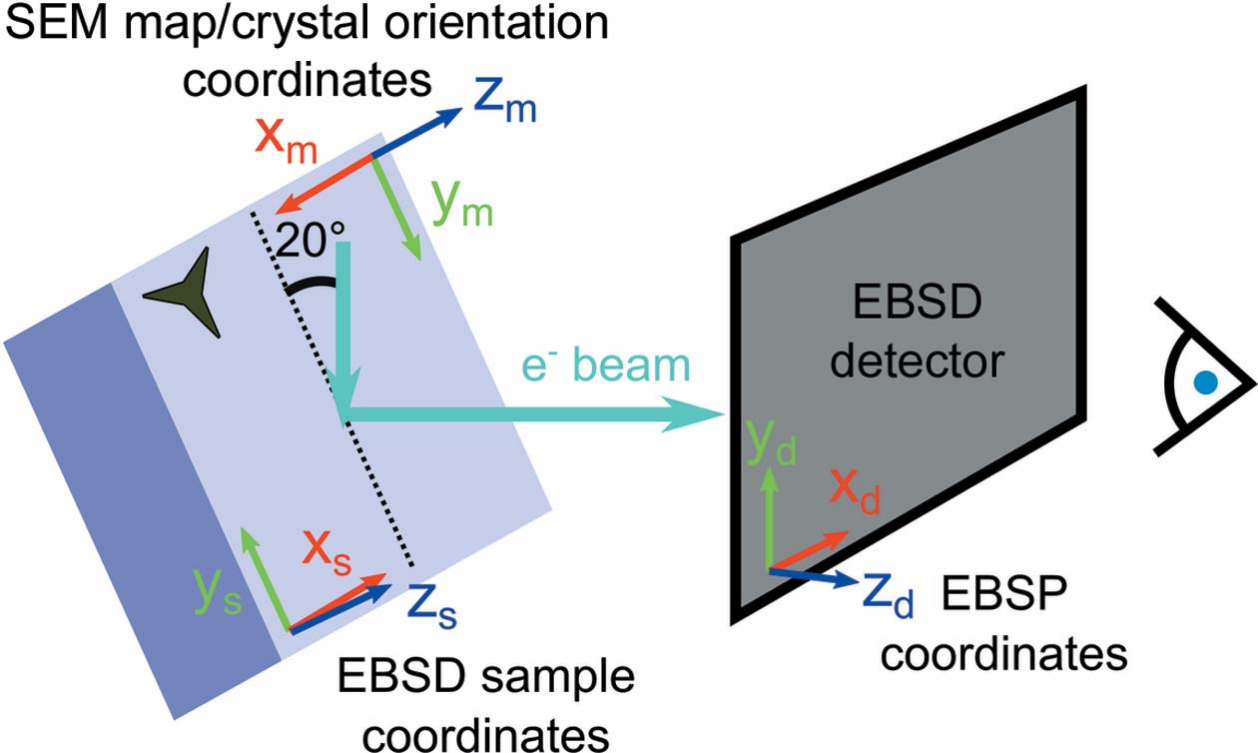
The position of the substrate in the laboratory frame for EBSD measurements, with relevant coordinate systems. The three-pointed star represents the orientation of a sample feature on the blue substrate. The EBSD detector coordinate system (*x*_d_, *y*_d_, *z*_d_) describes the EBSP coordinates. The EBSD sample coordinates (*x*_s_, *y*_s_, *z*_s_) correspond to the EBSD scan points. The SEM map coordinates (*x*_m_, *y*_m_, *z*_m_) show how EBSPs overlay on SEM maps. Here, the Euler angles output by the EBSD software are with respect to the SEM map coordinates. This follows the same convention as Britton *et al.* (2016 ▸).

Crystal orientation was determined by EBSD using a ZEISS Merlin instrument equipped with a Bruker Quantax EBSD system and a Bruker eFlash detector tilted at 4°. Electron backscatter patterns (EBSPs) were recorded with the sample tilted at 70° (Fig. 4 ▸) using an accelerating voltage of 30 kV and a current of 15 nA. The EBSPs were 800 × 600 pixels and a step size of 19.8 nm was used between consecutive points on the sample. The diffraction patterns were indexed and the Euler angles extracted for each pattern using the Bruker *ESPRIT 2.1* EBSD software. The Euler angles were exported and analysed using *MTEX*, a MATLAB toolbox for texture analysis (Bachmann *et al.*, 2011 ▸), to produce inverse pole figure (IPF) maps for all crystals (Fig. 2 ▸).

Here we use the Bunge convention (Bunge, 1982 ▸) to describe each crystal orientation (crystal frame) relative to the substrate (sample frame). The crystal orientation matrix **UB** is composed of **U**, which describes the rotation of the crystal reference frame, and **B** [equation (2)], which characterizes the unit-cell parameters.

Using the same convention as Britton *et al.* (2016 ▸), the unit cell has vectors **a**, **b** and **c** with lengths *a*, *b* and *c*, respectively. Angle α describes the angle between **b** and **c**, β the angle between **c** and **a**, and γ the angle between **a** and **b**. **B** is used to transform the base vectors to Cartesian base vectors: 

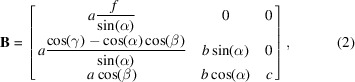

where 

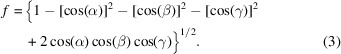




Since all crystals in this study have an f.c.c. structure, **B** is the 3 × 3 identity matrix multiplied by the lattice constant.

**UB** provides the direction and radial position of specific *hkl* reflections, **H**_*hkl*_, in laboratory coordinates (Busing & Levy, 1967 ▸), 

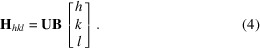




Several different coordinate frames are used in EBSD that refer to different aspects of the measurement (Fig. 4 ▸). In this paper, all coordinate systems and rotation matrices will be right handed and we will use the same notation as Britton *et al.* (2016 ▸). For EBSD, the following subscripts describe specific coordinate systems:

(i) ‘d’ is the detector frame that describes the EBSPs.

(ii) ‘s’ is the EBSD sample frame that is related to the detector frame by sample (θ_sample_) and detector (θ_detector_) tilts about the *x* axis. The *x*_s_ and *y*_s_ axes correspond to the directions of EBSD scan points.

(iii) ‘m’ is the SEM map frame. This corresponds to how the EBSPs overlay on the SEM maps and thus how the EBSD orientation is referenced.

The EBSD software returns the orientation matrix from EBSD measurements in the SEM map frame (Fig. 4 ▸). The corresponding orientation matrix is referred to as **U**_EBSD, m_. It can be constructed from a series of rotations using Euler angles, where each angle describes a rotation about a coordinate axis. Here we use right-handed rotation matrices to describe vector rotations about the *x* axis, 

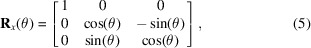

and the *z* axis, 

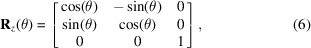

and Bunge-convention Euler angles, ϕ_1_, Φ and ϕ_2_ (Britton *et al.*, 2016 ▸). Equivalently, equations (5) and (6) correspond to left-handed rotations through angle θ for the coordinate system. To transform vectors from the crystal coordinate frame to the laboratory frame, we use (Britton *et al.*, 2016 ▸)

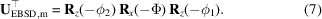




We note that the Euler angles in equation (7) are negative because the original angles as defined are for left-handed rotation matrices. First a rotation of −ϕ_1_ is applied about the original *z* axis, followed by a rotation of −Φ about the new *x* axis and finally a rotation of −ϕ_2_ about the new *z* axis. For consistency with the convention used here (Busing & Levy, 1967 ▸), we express equation (7) as 

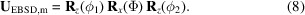

Here, the EBSD software already accounts for the instrument tilts θ_sample_ and θ_detector_ and returns Euler angles in the SEM map frame (subscript m) (Fig. 4 ▸).

To input the orientation matrix into the *spec* orientation calculator on beamline 34-ID-C, we must define two *hkl* reflections corresponding to out-of-plane [equation (13)] and in-plane [equation (14)] reflections (Hofmann *et al.*, 2017*a* ▸). One concern is the consistency in the indexing of crystals on the 34-ID-E Laue instrument and in EBSD measurements. Due to the cubic structure of the present crystals, there are equivalent orientation matrices that differ by 90° rotations about the crystal axes. These rotations are accounted for using an **R**_crystal_ rotation matrix that captures rotations by 90(*n*_*x*, *y*, *z*_)°, where *n*_*x*, *y*, *z*_ ∈ {−1, 0, 1, 2}, about the *x*, *y* and *z* axes, 

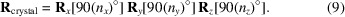




**Figure 5:**
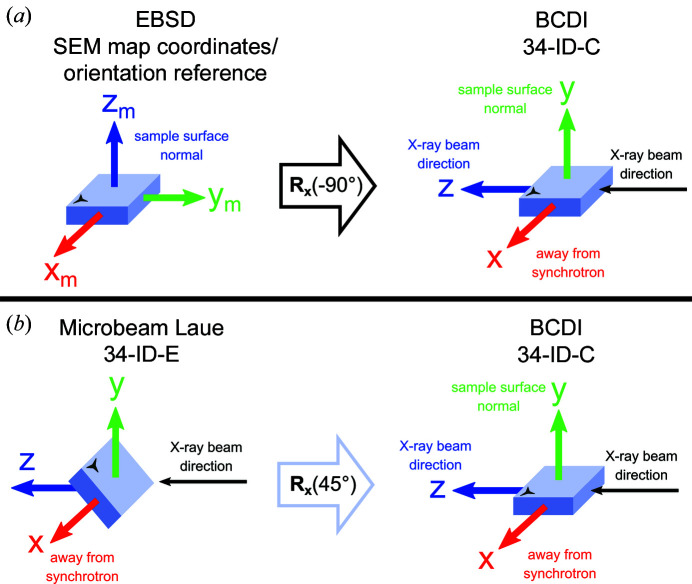
The position of the substrate in the laboratory frame for microbeam Laue diffraction and EBSD measurements compared with the BCDI laboratory frame (*x*, *y*, *z*). The three-pointed star represents the orientation of an arbitrary sample feature on the blue substrate. Right-handed rotation matrices are used for the rotations. (*a*) Diagram showing how the EBSD laboratory coordinates, specifically the SEM map coordinates (Fig. 4 ▸), are related to the BCDI frame. (*b*) Diagram showing the transformation between the laboratory frames for Laue diffraction and BCDI, characterized by a 45° rotation about the *x* axis.

To align the EBSD map frame to the BCDI laboratory frame, a −90° rotation about the *x* axis is required [Fig. 5 ▸(*a*)]. Combining this with equations (8) and (9) leads to the formation of the **UB**_34C, EBSD_ matrix,

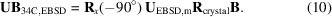

These matrix-based orientation operators provide a generalized approach to the transformation of orientation, irrespective of the software implementation.

### 2.3. Microbeam Laue X-ray diffraction

Microbeam Laue diffraction was used to verify the lattice orientation of each crystal independently. This was performed on beamline 34-ID-E at the APS. Further details about the instrument can be found elsewhere (Liu *et al.*, 2004 ▸; Hofmann *et al.*, 2017*a* ▸).

The sample was positioned with its surface inclined at a 45° angle to the incident beam [see Fig. 5 ▸(*b*)] and diffraction patterns were recorded using a Perkin–Elmer flat-panel detector above the sample. 2D fluorescence measurements of the Fe *K*α_1_ peak (6.40 keV) were used to identify the spatial position of the crystals, using a monochromatic 17 keV (Δλ/λ ≃ 10^−4^) X-ray beam focused to 0.25 × 0.25 µm (horizontal × vertical) using Kirkpatrick–Baez (KB) mirrors.

**Figure 6:**
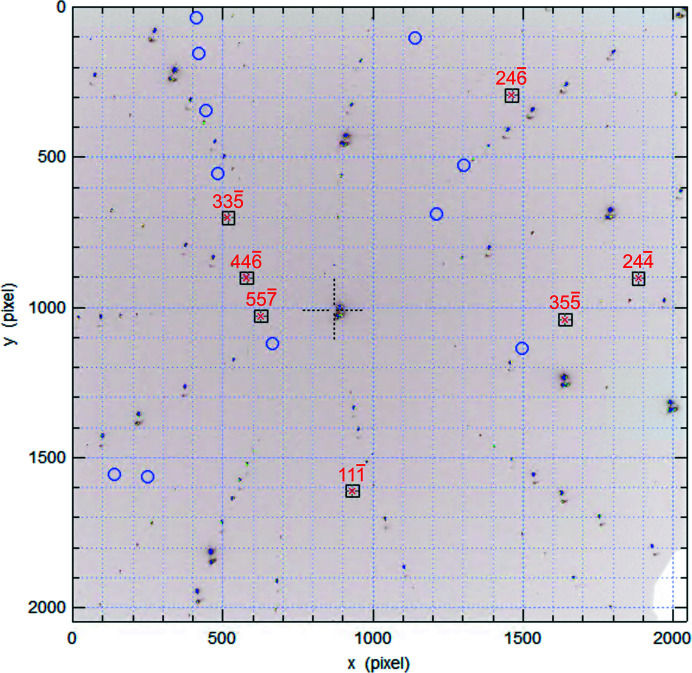
The Laue diffraction pattern from microcrystal 1B. The squares show the Bragg peaks that were used for orientation determination, with their corresponding *hkl* indices shown in red. Weaker reflections from the microcrystal are expected to be inside the blue circles. Other intense peaks in the Laue diffraction pattern belong to the sapphire substrate and are not indexed here for clarity.

Next, a polychromatic X-ray beam was used to collect a Laue diffraction pattern of each crystal (Fig. 6 ▸). The pattern shows weak Bragg reflections from the microcrystals and strong Bragg peaks from the single-crystal sapphire substrate. The two sets of peaks were indexed and fitted using the *LaueGo* software (https://www.aps.anl.gov/Science/Scientific-Software/LaueGo). From the indexing, we could determine the **UB** matrix [equation (4)]. The **UB** matrix determined by Laue diffraction on the 34-ID-E instrument is referred to as **UB**_Laue_, 

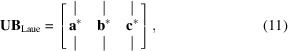

where a*, b* and c* are the column (represented by vertical lines) reciprocal-space vectors returned by *LaueGo* in units of nm^−1^ in the 34-ID-E laboratory frame.

To convert **UB**_Laue_ into a **UB** matrix for use on the BCDI instrument 34-ID-C, **UB**_34C, Laue_, the 45° rotation of the sample in the Laue laboratory frame must be accounted for [Fig. 5 ▸(*b*)]. To align the microbeam Laue and BCDI laboratory frames, a rotation of the sample by 45° about the *x* axis is required (Hofmann *et al.*, 2017*a* ▸), leading to 

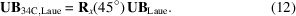




### 2.4. Bragg coherent X-ray diffraction imaging

BCDI was performed on beamline 34-ID-C at the APS. An *in situ* confocal microscope was used to position the microcrystal within the X-ray beam (Beitra *et al.*, 2010 ▸). The sample was illuminated using a 9 keV (λ = 0.138 nm) coherent X-ray beam, with a bandwidth of Δλ/λ ≃ 10^−4^, from an Si(111) monochromator. The X-ray beam was focused to a size of 1.1 × 1.1 µm (horizontal × vertical, FWHM) using KB mirrors. Beam-defining slits were used to select the coherent portion of the beam at the entrance to the KB mirrors. For beamline 34-ID-C, the transverse coherence length is ξ_h_ > 10 µm and the longitudinal coherence length is ξ_w_ ≃ 0.7 µm at a photon energy of 9 keV (Leake *et al.*, 2009 ▸).

The sample needs to be positioned such that a specific *hkl* Bragg diffraction condition is met to produce a diffraction pattern in the far-field Fraunhofer regime. The orientation matrix determined by EBSD or Laue diffraction is communicated to the *spec* software used on 34-ID-C by defining two reflections that correspond to *hkl* values associated with laboratory **x** (in-plane) and **y** (out-of-plane) directions [note the angles referred to here are set elsewhere (Hofmann *et al.*, 2017*a* ▸)]:

(i) The primary reflection (out-of-plane, **y** direction), **H**_⊥_, where the instrument angles are set to δ_*spec*_ = 0°, γ_*spec*_ = 20°, θ_*spec*_ = 0°, χ_*spec*_ = 90° and ϕ_*spec*_ = −10°. The fractional 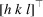 is then 

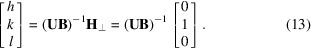




(ii) The secondary reflection (in-plane, **x** direction), **H**_∥_, where the instrument angles were set to δ_*spec*_ = 20°, γ_*spec*_ = 0°, θ_*spec*_ = 10°, χ_*spec*_ = 90° and ϕ_*spec*_ = 0°. The fractional 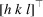 is then 

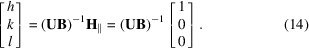




Here **UB** refers to **UB**_34C, EBSD_ or **UB**_34C, Laue_. These two fractional *hkl* vectors are then entered into *spec* as known reflections. On this basis, the expected angular positions of the {111} and {200} reflections from the sample were calculated. Not all {111} and {200} reflections could be measured as some may exceed the angular range of the sample and detector motors. Occasionally, manual motor adjustments of a few degrees were required to locate the Bragg peak. Once a Bragg peak was found, the sample tilt and positioning were refined such that the centre of mass was on the centre of the detector positioned 0.5 m away from the sample. At this aligned position, the angular and translational positions were saved for each Bragg peak and used to determine the true beamline orientation matrix **UB**_34C_ by minimizing the least-squares error associated with the measured reflections, 

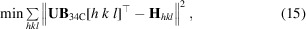

where 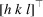 are the Miller indices in crystal coordinates.

Differences between the true and predicted positions of the reflections using **UB**_34C_ arise from a number of different sources. The largest error is the repeatability of the sample position in different coordinate systems. The use of Thorlabs 1X1 kinematic mounts, which have angular errors of less than a millidegree, helps with the precise re-mounting of samples. There is also uncertainty in the goniometer precision and alignment with the diffractometer, which can influence the angle readout. Furthermore, the centre of the detector may not be perfectly aligned to the calculated position for a given detector distance or angle. The position of the measured Bragg peak is limited by the energy resolution of the incident X-ray as it affects the Bragg angle.

CXDPs were collected on a 256 × 256 pixel module of a 512 × 512 pixel Timepix area detector (Amsterdam Scientific Instruments) with a GaAs sensor and a pixel size of 55 × 55 µm positioned 1.0 m from the sample to ensure oversampling. CXDPs were recorded by rotating the crystal through an angular range of 0.6° and recording an image every 0.005° with 0.1 s exposure time and 50 accumulations at each angle.

To optimize the signal-to-noise ratio and increase the dynamic range of the CXDPs, three repeated scans for each of the 111, 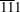 , 200, 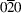 and 002 reflections were performed and aligned to maximize their cross correlation. Once aligned, the minimum acceptable Pearson cross correlation for summation of CXDPs from a specific Bragg reflection was chosen to be 0.976, similarly to previous BCDI studies (Hofmann *et al.*, 2018 ▸, 2020 ▸). CXDPs were corrected for dead time, dark field and white field prior to cross-correlation alignment. Details regarding the recovery of the real-space images using phase retrieval algorithms are given in Appendix *A* [App appa] and the computation of the strain is given in Appendix *B* [App appb].

### 2.5. Sample mounting

For the SEM, EDX and EBSD analyses, samples were mounted on 12.5 mm diameter SEM specimen pin stubs using silver paint. For microbeam Laue X-ray diffraction, they were attached to a Thorlabs 1X1 kinematic mount. From here, a Thorlabs kinematic mount adapter between 34-ID-E and 34-ID-C was used to mount the samples for BCDI. This adapter consists of two 1X1 mounts sandwiched together to enable sample orientation to be well preserved between the beamlines. There is no kinematic mount adapter between the SEM and BCDI instruments. Moreover, the use of magnets in the kinematic mounts inhibits their use for electron microscopy. Across the different instruments, the sample orientation was maintained throughout as shown in Fig. 5 ▸, which has an arbitrary sample feature to illustrate the respective orientations.

## 3. Results and discussion

### 3.1. Orientation matrix comparison

The angular mismatch between two **UB** matrices, **UB**_1_ and **UB**_2_, can be determined by converting **UB**_1_(**UB**_2_)^−1^ into a rotation vector. A rotation matrix **R** can be converted using Rodrigues’ rotation formula in matrix exponential form, 

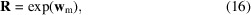

where **w**_m_ is an antisymmetric matrix, 

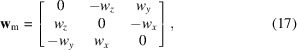

which contains the elements of the rotation vector 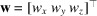 . The rotation vector is defined by a rotation axis 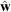 multiplied by a rotation θ. If the two orientation matrices are different, **UB**_1_(**UB**_2_)^−1^ can be converted to a rotation vector where the angular mismatch is θ. If **UB**_1_ = **UB**_2_, then **UB**_1_(**UB**_2_)^−1^ = *I*_3_ and therefore θ = 0.

Here we set **UB**_1_ and **UB**_2_ as **UB**_34C, EBSD_, **UB**_34C, Laue_ or **UB**_34C_. First we rearrange equation (16) to calculate **w**_m_: 

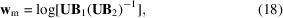

where 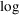 here refers to the matrix natural logarithm. Next we reconstruct the rotation vector using equation (17) and calculate its magnitude to obtain the mismatch, 

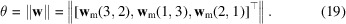




To calculate the angular mismatch between **UB**_34C, EBSD_ and other orientation matrices, the permutation of *n*_*x*, *y*, *z*_ that produced the smallest error was chosen. Tables 1 ▸–3 ▸ ▸ show the angular mismatch of **UB**_34C, Laue_ and **UB**_34C, EBSD_ compared with **UB**_34C_ for substrates 1–3. The average angular mismatch for all crystals between orientation matrices **UB**_34C, Laue_ and **UB**_34C_ is 4.48°, that between **UB**_34C, EBSD_ and **UB**_34C_ is 6.09°, and that between **UB**_34C, Laue_ and **UB**_34C, EBSD_ is 1.95°. Generally, **UB**_34C, Laue_ is most similar to **UB**_34C_. This is expected, as there is a Thorlabs kinematic mount adapter between 34-ID-E and 34-ID-C for the precise angular alignment of samples. A larger difference is observed when comparing **UB**_34C, EBSD_ and **UB**_34C_. This is due to the manual removal of the SEM pin stub from the electron microscope, which then needs to be re-secured to the kinematic mount. The cylindrical pin permits a greater degree of rotational freedom, thereby increasing the angular mismatch when **UB**_34C, EBSD_ is considered. Despite this increased angular freedom in the pin, a 2° increase in the angular uncertainty when using **UB**_34C, EBSD_ instead of **UB**_34C, Laue_ is still a very accurate result. This means only a slightly larger angular range needs to be explored in alignment.

The alignment of crystals for BCDI using EBSD remains much more time efficient because microbeam Laue diffraction pre-alignment on 34-ID-E is no longer required. The use of EBSD for pre-alignment of BCDI samples also affords greater flexibility in experiment type. The ability to pre-characterize samples offsite should substantially increase throughput and make MBCDI a much more widely accessible technique, especially on beamlines without pink-beam capability or access to a nearby Laue instrument.

### 3.2. Determination of strain

**Figure 7:**
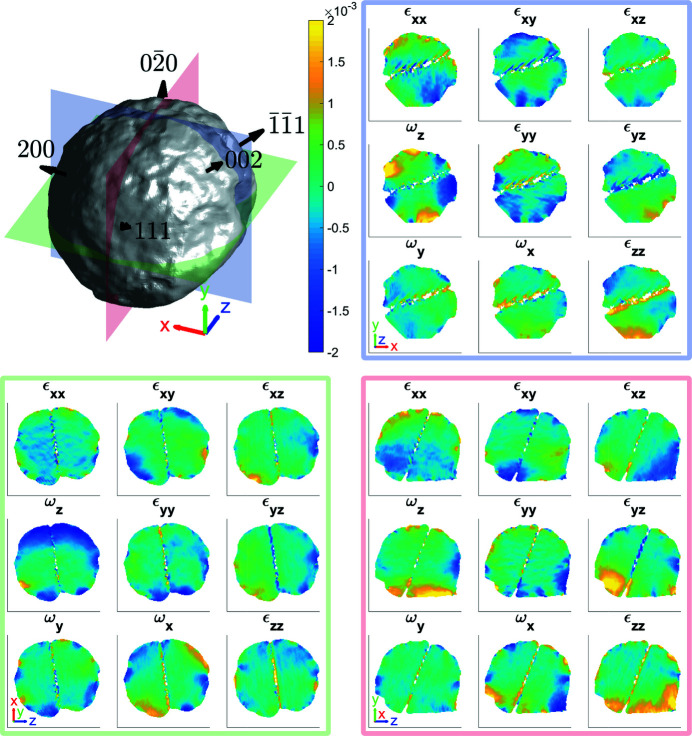
The average morphology of the 111, 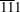 , 200, 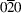 and 002 reflections for Fe–Ni crystal 1B. The region of missing intensity in the middle of the crystal corresponds to a twinned region of the crystal, which is only visible in the 111 reconstruction (Fig. 10 in Appendix *A* [App appa]). The average morphology taken over all five reflections is based on an amplitude threshold of 0.30. The slices through the strain and rotation tensor components using the average morphology are shown for the planes indicated at *x* = 2.5 nm (red), *y* = 2.5 nm (green) and *z* = 2.5 nm (blue) from the centre of mass of the microcrystal. The amplitude threshold is 0.30 and the coordinate axes are 100 nm long. The supplementary videos (SV1–SV3) show the strain and rotation tensor components throughout the volume along the *x*, *y* and *z* axes, respectively.

MBCDI allows the strain and rotation tensors to be computed if at least three reflections are measured, thus providing more information about the crystal defects present (Hofmann *et al.*, 2017*b* ▸, 2018 ▸, 2020 ▸; Phillips *et al.*, 2020 ▸). Fig. 7 ▸ shows the strain and rotation tensors reconstructed from five measured Bragg reflections of crystal 1B. The ε_*xx*_, ε_*yy*_ and ε_*zz*_ slices show defects close to the edge that may not be resolved if analysing a single reflection alone (Yang *et al.*, 2021 ▸), as some crystal defects, such as dislocations, are visible only when **Q**_*hkl*_ · **b** ≠ 0, where **b** is the Burgers vector (Williams & Carter, 2009 ▸).

These results were produced using a refined method for the computation of the strain and rotation tensor. A general approach for the computation of both these tensors is described in Appendix *B* [App appb]. This relies on the recovery of the phase of the CXDP. The intensity of the CXDP is the squared magnitude of the Fourier transform 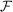 of the complex crystal electron density *f*. The solution for the recovered phase is non-unique, as global phase offsets *C* can produce the same CXDP, *i.e.* 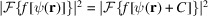 . After phase retrieval, the phase values are bound between [−π, π], which describes the periodic nature of the crystal structure but not necessarily the true complex crystal electron density. For instance, if the projected displacement in the direction of **Q**_*hkl*_ is greater than π/|**Q**_*hkl*_|, then a phase jump, where the phase difference is 2π between two pixels, will occur. These phase jumps cause discontinuities in the derivatives of the phase ∂ψ_*hkl*_(**r**)/∂*j*, where *j* corresponds to the spatial *x*, *y* or *z* coordinate, leading to spurious large strains. Typically, phase unwrapping algorithms can be used to remove phase jumps, but dislocations have characteristic phase vortices (Clark *et al.*, 2015 ▸) that end at dislocation lines, meaning that phase jumps associated with dislocations cannot be unwrapped.

To account for this, Hofmann *et al.* (2020 ▸) demonstrated an approach that involves producing two additional copies of the phase with phase offsets of 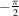 and 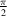 , respectively. This shifts the phase jumps to different locations and, by choosing the phase gradient with the smallest magnitude for each voxel, the correct phase derivatives can be found. Here, we employ a more efficient method used in coherent X-ray diffraction tomography following Guizar-Sicairos *et al.* (2011 ▸), which has also been applied in the Bragg geometry using ptychography (Li *et al.*, 2021 ▸). Rather than making multiple copies of the phase, we take the derivative of the complex exponential of the phase and determine the phase gradient using the chain rule: 

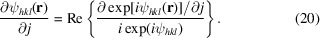

**Figure 8:**
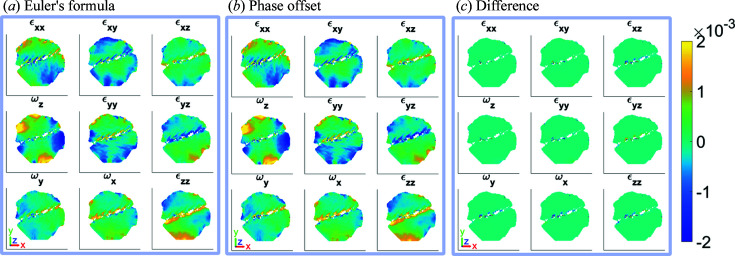
A comparison of the strain and rotation tensors at *z* = 2.5 nm, as shown in Fig. 7 ▸, computed using two different methods. (*a*) The strain and rotation tensors as computed using equation (20) (Guizar-Sicairos *et al.*, 2011 ▸). (*b*) The strain and rotation tensors as computed by introducing phase offsets and choosing the phase gradient with the minimum value (Hofmann *et al.*, 2020 ▸). (*c*) The difference between the results. The amplitude threshold for the reconstructions is 0.30 and the magnitude of the coordinate axes is 100 nm.

Here 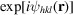 is a circle expressed using Euler’s formula, where the phase jumps disappear. Fig. 8 ▸ shows the difference between the two methods for computing the lattice rotation and strain tensors. We can see that the procedure based on phase offsets (Hofmann *et al.*, 2020 ▸) fails to resolve the details fully in the regions with high strain, *i.e.* around the edges and central region of missing intensity. The new approach of computing phase gradients successfully deals with these complex regions.

**Figure 9:**
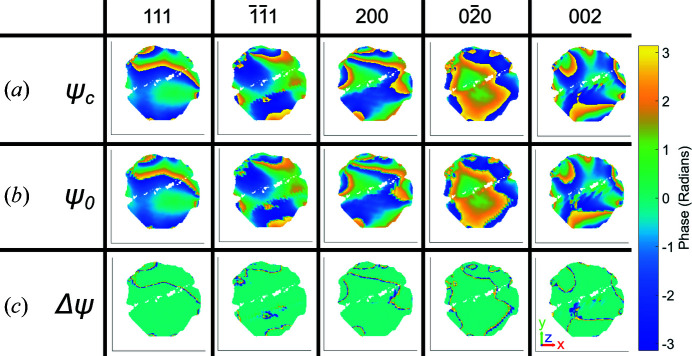
Phases for the 111, 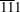 , 200, 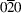 and 002 reflections at *z* = 2.5 nm. We compare (*a*) the new method of interpolating the complex quantity of the phase ψ_c_ with (*b*) interpolating the phase alone ψ_0_. (*c*) The difference between ψ_c_ and ψ_0_, Δψ, highlights the information that can only be resolved around phase jumps in ψ_c_. The amplitude threshold for the reconstructions is 0.30 and the size of the coordinate axes is 100 nm. Here the average morphology was used. Hence the 111 reconstruction shows a region of missing intensity corresponding to a twin, which is not present in the original 111 reconstructed morphology (Fig. 10 ▸ in Appendix *A* [App appa]).

Furthermore, we apply this to the interpolation of the phase from detector conjugated space to sample space, by interpolating the complex quantity 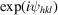 instead of ψ_*hkl*_. This avoids the blurring of phase jumps that occurs during direct interpolation of ψ_*hkl*_, shown in Fig. 9 ▸.

This refinement of strain and rotation tensor computation allows for a more accurate reconstruction of crystal defects and their associated nanoscale lattice strains. It also reduces the time required to compute the tensors, since phase offsets need to be applied before and after mapping the crystal from detector conjugated space to orthogonal sample space. This will play an important role in the analysis of large MBCDI data sets, such as those obtained from *in situ* or *operando* experiments that reveal crystal defects interacting with their environment. Reconstruction accuracy in multi-Bragg-peak phase retrieval algorithms that involve a shared displacement field constraint applied to all reflections would also be improved by implementing this more accurate interpolation of phase values (Newton, 2020 ▸; Gao *et al.*, 2021 ▸; Wilkin *et al.*, 2021 ▸).

## 4. Conclusions

We have demonstrated that the orientation of various microcrystals on different substrates can be found via EBSD and used to align BCDI experiments. The results indicate a ∼2° increase in angular error when using EBSD alignment compared with Laue diffraction alignment, which is still within reasonable tolerance for the search for Bragg peaks. Importantly, using EBSD to pre-align crystals allows beamtime to be more effectively utilized for BCDI data set collection. It also removes the need for BCDI and Laue instrument coordination, and enables MBCDI on BCDI instruments that do not have pink-beam capability or a Laue instrument nearby. Using the orientation matrix obtained from EBSD, five reflections have been located on an Fe–Ni microcrystal and full 3D strain and rotation tensors have been recovered. When computing the tensors, we have demonstrated a more efficient approach to resolving phase jumps, by implementing a complex phase quantity to calculate and interpolate the phase. This allows for the phase to be unwrapped and the correct strain to be resolved in the vicinity of dislocations. These refinements make BCDI a more accessible microscopy tool.

## 5. Data availability

The processed diffraction patterns, final reconstructions and data analysis scripts, including a script to compute the orientation matrix using EBSD, are publicly available at https://doi.org/10.5281/zenodo.6383408.

Also available are three supplementary videos, as follows:

(i) SV1 shows *xy* plane slices through the lattice strain and rotation tensors in Fig. 7 ▸.

(ii) SV2 shows *yz* plane slices through the lattice strain and rotation tensors in Fig. 7 ▸.

(iii) SV3 shows *zx* plane slices through the lattice strain and rotation tensors in Fig. 7 ▸.

## Supplementary Material

The processed diffraction patterns, final reconstructions and data analysis scripts, including a script to compute the orientation matrix using EBSD: https://doi.org/10.5281/zenodo.6383408

Supplementary video SV1. DOI: 10.1107/S1600576722007646/te5099sup1.mp4

Supplementary video SV2. DOI: 10.1107/S1600576722007646/te5099sup2.mp4

Supplementary video SV3. DOI: 10.1107/S1600576722007646/te5099sup3.mp4

## Figures and Tables

**Figure 10:**
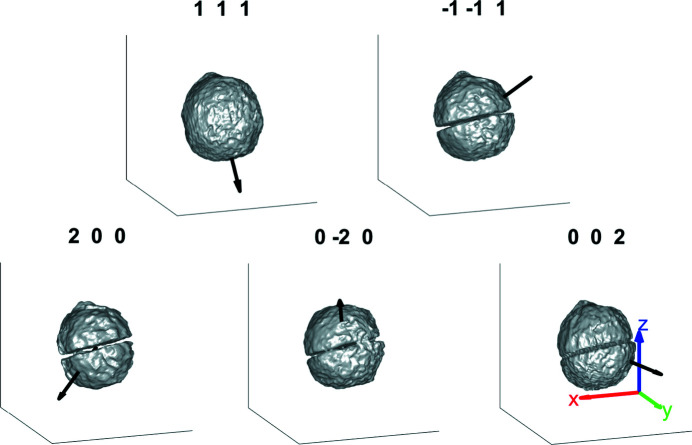
The sample morphology for each measured reflection labelled with the scattering vector. The average morphology for the five reflections is shown in Fig. 7 ▸. The amplitude threshold for the reconstructions is 0.30 and the size of the coordinate axes is 500 nm.

**Figure 11:**
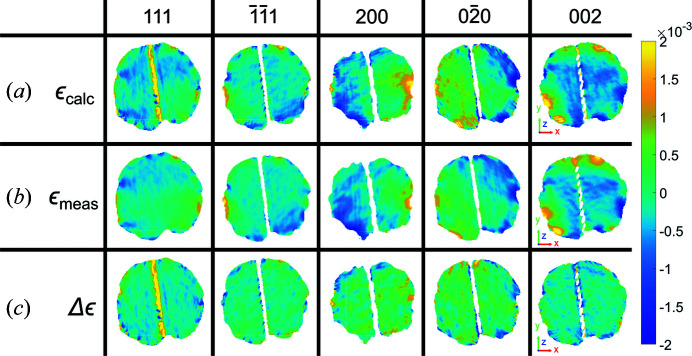
A comparison between (*a*) the calculated and (*b*) the measured strain for the 111, 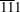 , 200, 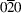 and 002 reflections at *y* = 2.5 nm. The calculated strain is computed from the strain tensor determined using the other four reflections following the methodology presented in the text. (*c*) The measured strain subtracted from the calculated strain. The amplitude threshold for the reconstructions is 0.30 and the size of the coordinate axes is 100 nm.

**Figure 12:**
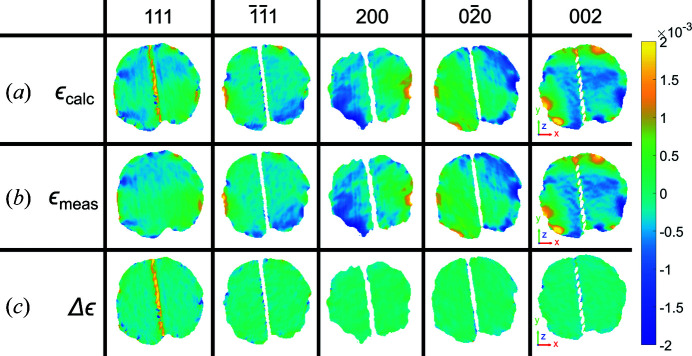
A comparison between (*a*) the calculated and (*b*) the measured strain for the 111, 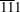 , 200, 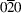 and 002 reflections at *y* = 2.5 nm. The calculated strain is computed using the strain tensor determined using all five reflections following the methodology presented in the text. (*c*) The measured strain subtracted from the calculated strain. The amplitude threshold for the reconstructions is 0.30 and the size of the coordinate axes is 100 nm.

**Table 1:** Angular differences (°) between **UB**_34C_, **UB**_34C, Laue_ and **UB**_34C, EBSD_ for substrate 1

Sample		**UB**_34C_	**UB**_34C, Laue_	**UB**_34C, EBSD_
1B	**UB**_34C_	–	–	–
	**UB**_34C, Laue_	9.72	–	–
	**UB**_34C, EBSD_	11.0	2.37	–

1C	**UB**_34C_	–	–	–
	**UB**_34C, Laue_	2.22	–	–
	**UB**_34C, EBSD_	5.35	3.28	–

1E	**UB**_34C_	–	–	–
	**UB**_34C, Laue_	9.70	–	–
	**UB**_34C, EBSD_	11.0	1.97	–

1F	**UB**_34C_	–	–	–
	**UB**_34C, Laue_	7.14	–	–
	**UB**_34C, EBSD_	9.56	3.05	–

Average	**UB**_34C_	–	–	–
	**UB**_34C, Laue_	7.19	–	–
	**UB**_34C, EBSD_	9.23	2.67	–

**Table 2:** Angular differences (°) between **UB**_34C_, **UB**_34C, Laue_ and **UB**_34C, EBSD_ for substrate 2

Sample		**UB**_34C_	**UB**_34C, Laue_	**UB**_34C, EBSD_
2A	**UB**_34C_	–	–	–
	**UB**_34C, Laue_	1.81	–	–
	**UB**_34C, EBSD_	2.00	0.845	–

2B	**UB**_34C_	–	–	–
	**UB**_34C, Laue_	0.366	–	–
	**UB**_34C, EBSD_	3.33	3.56	–

2C	**UB**_34C_	–	–	–
	**UB**_34C, Laue_	11.0	–	–
	**UB**_34C, EBSD_	11.2	0.306	–

2D	**UB**_34C_	–	–	–
	**UB**_34C, Laue_	0.392	–	–
	**UB**_34C, EBSD_	0.659	0.269	–

2E	**UB**_34C_	–	–	–
	**UB**_34C, Laue_	0.977	–	–
	**UB**_34C, EBSD_	4.34	4.11	–

2F	**UB**_34C_	–	–	–
	**UB**_34C, Laue_	0.425	–	–
	**UB**_34C, EBSD_	1.58	1.23	–

Average	**UB**_34C_	–	–	–
	**UB**_34C, Laue_	2.49	–	–
	**UB**_34C, EBSD_	3.86	1.52	–

**Table 3:** Angular differences (°) between **UB**_34C_, **UB**_34C, Laue_ and **UB**_34C, EBSD_ for substrate 3

Sample		**UB**_34C_	**UB**_34C, Laue_	**UB**_34C, EBSD_
3A	**UB**_34C_	–	–	–
	**UB**_34C, Laue_	0.369	–	–
	**UB**_34C, EBSD_	2.28	2.25	–

3B	**UB**_34C_	–	–	–
	**UB**_34C, Laue_	9.69	–	–
	**UB**_34C, EBSD_	10.7	2.09	–

Average	**UB**_34C_	–	–	–
	**UB**_34C, Laue_	5.03	–	–
	**UB**_34C, EBSD_	6.49	1.74	–

## Appendix A Phase retrieval

The reconstruction process of CXDPs was done independently for each reflection, in two stages, using the output from the previous stage to seed the next phasing stage (listed below). The CXDPs have a size of 256 × 256 × 128 voxels.

(i) Each reconstruction was seeded with a random guess. A guided phasing approach (Chen *et al.*, 2007 ▸) with 40 individuals and four generations was used with a geometric average breeding mode. For each generation and population, a block of 20 error reduction (ER) and 180 hybrid input–output (HIO) iterations, with β = 0.9, was repeated three times. This was followed by 20 ER iterations to return the final object. The shrinkwrap algorithm (Marchesini *et al.*, 2003 ▸) with a threshold of 0.1 was used to update the real-space support after every iteration. The σ values for the Gaussian kernel shrinkwrap smoothing for each generation were σ = 2.0, 1.5, 1.0 and 1.0, respectively. The best reconstruction was determined using a sharpness criterion, as it is the most appropriate metric for crystals containing defects (Ulvestad *et al.*, 2017 ▸). The two worst reconstructions were removed after each generation.

(ii) The reconstruction was seeded with the output from stage (i). This second stage was identical to stage (i), except now for each generation and population a block of 20 ER and 180 HIO iterations, with β = 0.9, was repeated 15 times, followed by 1000 ER iterations. Here the final 50 iterates were averaged to produce the final image.

The overall average 3D spatial resolution was 35 nm. This was determined by differentiating the electron-density amplitude across the crystal/air interface for the five reflection directions and fitting a Gaussian to each of the profiles. The reported spatial resolution is the averaged FWHM of the Gaussian profiles. The average 3D spatial resolutions were 30, 38, 39, 31 and 39 nm for the 111,  , 200,  and 002 reconstructions (Fig. 10 ▸), respectively.

## Appendix B Strain and rotation tensor calculations

The full 3D lattice strain tensor ɛ(**r**) and rotation tensor ω(**r**) are given by (Constantinescu & Korsunsky, 2007 ▸)

and

which rely on the reconstruction of **u**(**r**). With a single BCDI measurement, we can determine one component of the displacement field. For MBCDI, if at least three linearly independent reflections are measured, **u**(**r**) can be determined by minimizing the least-squares error (Hofmann *et al.*, 2017*a* ▸; Newton *et al.*, 2010 ▸),

for every voxel in the sample. Here, we use the modified approach of Hofmann *et al.* (2020 ▸), in which case the squared error between phase gradients is minimized,

where *j* corresponds to the spatial *x*, *y* or *z* coordinate, to find ∇**u**(**r**) directly for the computation of ɛ(**r**) and ω(**r**) in equations (21) and (22), respectively.

In Fig. 7 ▸, five linearly independent reflections were measured and reconstructed to assemble the strain and rotation tensor. To assess the reliability of the MBCDI measurements and phase retrieval procedure, we calculate the strain tensor using four out of the five reflections to predict the strain projected along the scattering vector of the fifth reflection. First, the phase gradients for each reflection are calculated from the strain tensor components,

and then used to calculate the strain fields projected along the scattering vector ε_*hkl*_(**r**),

This is compared with the strain field projected along the scattering vector computed from the measured phase gradients using the approach presented in the main text [equation (20)].

The average strain error for each reflection is computed by summing the magnitude of the difference between the calculated and measured strain and dividing the sum by the number of voxels in the morphology of each reconstruction (Fig. 11 ▸). The average strain error for each reflection (listed in parentheses) is 3.5 × 10^−4^ (111), 2.1 × 10^−4^ (  ), 3.3 × 10^−4^ (200), 4.0 × 10^−4^ (  ) and 2.9 × 10^−4^ (002).

If all five reflections are used to predict the strain along the scattering vector for each reflection (Fig. 12 ▸), the average strain error for each reflection (listed in parentheses) is 2.0 × 10^−4^ (111), 1.2 × 10^−4^ (  ), 8.2 × 10^−5^ (200), 1.0 × 10^−4^ (  ) and 9.6 × 10^−5^ (002).

The average strain error is close to the strain resolution limit for BCDI of ∼2 × 10^−4^ (Yang *et al.*, 2021 ▸; Hofmann *et al.*, 2020 ▸), demonstrating that the reconstructed tensors using all five reflections are accurate. The use of all five reflections reduces the average strain error by 62% compared with the use of four reflections.
